# Management of patients with chronic cough using a clinical protocol: a prospective observational study

**DOI:** 10.1186/1745-9974-9-2

**Published:** 2013-01-24

**Authors:** Josephine C Ojoo, Caroline F Everett, Siobhain A Mulrennan, Shoaib Faruqi, Jack A Kastelik, Alyn H Morice

**Affiliations:** 1 Department of Cardiovascular and Respiratory Studies, Castle Hill Hospital, University of Hull, Cottingham, HU16 5JQ, UK

**Keywords:** Cough, Differential diagnosis, Diagnostic techniques and procedures

## Abstract

**Background and aims:**

Chronic cough is a common symptom the aetiology of which can be challenging to diagnose. Diagnostic protocols for chronic cough have required the use of specialist investigations which are not always easily available. We wanted to determine whether patients with chronic cough can be successfully managed using a clinical algorithm.

**Methods:**

112 consecutive patients with chronic cough were prospectively recruited into this study. They were assessed by history, physical examination, chest radiograph, spirometry and reversibility to nebulised salbutamol. A clinical diagnosis was made and the patient had an 8-week trial of appropriate therapy. Further therapeutic trials were carried out depending on response to treatment and the possible differential diagnoses. Investigations were carried out in cases of failed clinical trials and to exclude specific pathology. The “clinical arm” comprised patients managed on the basis of clinical assessment and without any investigations. The “investigative arm” comprised those who needed further investigations.

**Results:**

81 (72%) were managed in the clinical arm. Of these 74 (66%) were discharged following response to therapy. 31 (28%) patients were converted to the investigative arm after failure of diagnosis in the clinical protocol. The commonest causes of cough were gastroesophageal reflux, asthma and chronic rhinitis. 51 (45.5%) patients responded to therapy based on diagnosis at initial assessment while a further 23 (20.5%) patients responded to sequential clinical trials for the commonest causes of cough. Cough severity score improved by a mean of 3.6 points on a numeric response score (from 0–10, p < 0.0001).

**Conclusion:**

It is possible to manage a majority of chronic cough patients successfully using a protocol based on presenting symptoms and therapeutic trials for the common causes of cough.

## Introduction

Chronic cough has been arbitrarily defined as a cough lasting more than 8 weeks [1]. The distinction between acute and chronic cough is important as the epidemiology and the aetiology of these differ. Chronic cough is a very common symptom with which patients present to both primary and secondary care [2,3]. A diagnostic approach involving the systematic use of specialist investigations to elucidate the underlying cause of the cough has been advocated [4]. Whilst this approach has been shown to result in successful diagnosis and treatment of a majority of chronic cough patients, it does require the routine use of investigations which may be uncomfortable for the patient and which may not be easily accessible to physicians in primary or secondary care [4,5].

Many studies from around the world have repeatedly confirmed that the commonest causes of chronic cough are gastro-oesophageal reflux (GOR), asthma and related syndromes and upper airways disease (post nasal drip syndrome, rhinitis and sinusitis) [6-8]. These diagnoses accounted for up to 93% of chronic cough in these studies. It is our experience that these conditions present with differentiated symptoms, which suggests that it should be possible to make a diagnosis from the history and examination.

Based on this premise we hypothesised that in a large proportion of patients with chronic cough the diagnosis can be made simply based on history, examination and simple tests that are commonly available and treatment of the underlying condition can be instituted in a timely fashion without undertaking expensive investigations. In cases where the diagnosis is not clear from initial assessment, sequential trial of therapy for the three commonest conditions should cause resolution of symptoms in a significant proportion of patients.

To test these hypotheses we designed a clinical protocol for the management of cough based on the history, examination, chest radiograph and spirometry tests, which would be available to all clinicians seeing patients with chronic cough. A protocol based on clinical assessment and routinely available simple tests would be particularly useful in primary care.

## Methods

### Subjects

Sequential consenting adult patients (112 patients, above 18 years of age) of either sex referred on both a secondary and tertiary basis to the Hull Cough Clinic with a history of chronic cough were prospectively enrolled into the study over a period of 18 months. Patients who had been on angiotensin converting enzyme (ACE) inhibitors had the therapy stopped at least one month prior to the clinic appointment. This is because ACE inhibitors cause a significant increase in the sensitivity of the cough reflex [9]. Thus, symptomatic cough due to any cause will be exacerbated by the concomitant use of an ACE inhibitor.

### Assessment

1. ***Clinical protocol***

**Figure 1:**
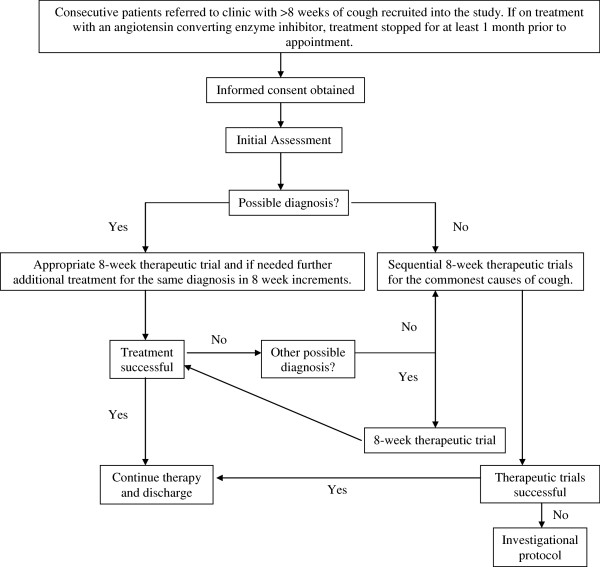
**Management pathway for the clinical protocol. **Figure legend: Initial empirical treatment for reflux related cough was acid suppressive therapy (lansoprazole 30 mg bd with ranitidine 300 mg on). If this did not lead to amelioration of symptoms and airway reflux was thought to be the cause, sequentially pro-kinetics (metoclopramide 10 mg tds / domperidone 10 mg tds) and baclofen (5 mg tds and increased to 10 mg tds depending upon response / tolerance) trials were instituted prior to transfer to sequential trials or the investigational protocol. The initial treatment for asthma and related disorders was with a high dose inhaled cortico-steroid and long acting beta2 agonist combination inhaler. Subsequent additional therapy was with montelukast (10 mg od). The treatment for rhinitis was with an inhaled nasal cortico-steroid with the addition of anti-histamine as additional therapy.

1.1 A detailed history and examination was carried out by one of the physicians (Cough Clinic Proforma, Additional file 1). The examination included complete general physical and systemic examinations.

1.2 Plain postero-anterior chest radiograph was performed in all patients at the time of the first visit to clinic. The film was assessed initially by the physician in clinic, but a formal report from a radiologist was also routinely obtained. This is a recommendation endorsed by all current guidelines in order to exclude serious lung pathology as a cause [1,5,10].

1.3 Lung function tests (spirometry and reversibility testing) were performed using a Vitalograph compact spirometer (Vitalograph Ltd®, Buckingham, UK) in order to screen for any significant airways disease. Parameters measured were forced expiratory flow volume in 1 second (FEV_1_) and forced vital capacity (FVC). The patient performed three forced expiratory manoeuvres from total lung capacity to residual volume and the best of the three was used in the analysis. To assess reversibility, we measured the percentage change in FEV_1_ 20 minutes after the administration of 2.5 mg of nebulised salbutamol (Ventolin Nebules® Allen and Hanburys Ltd, Middlesex, UK) over 5 minutes using an oxygen driven venturi mask.

1.4 The physician used this information to make a list of differential diagnoses and the patient was managed as outlined in Figure 1. Features considered to support the diagnosis of asthma were associated wheeze, breathlessness and an obstructive pattern or reversibility on spirometry. Presence of dyspeptic symptoms was considered to support a diagnosis of reflux related cough. A history of post nasal mucus drip and / or recurrent throat clearing would be supportive of a diagnosis of upper airways disease. The above were considered as general guides to diagnosis which was based on the overall assessment of the individual clinician. An 8-week trial of therapy for the main diagnosis was carried out after which the patient was reviewed for response. If there had been no improvement then either treatment for the initially made diagnosis was further escalated or empirical treatment for the next likely cause of cough was instituted. If history and examination suggested more than one cause of cough and there was partial improvement of the cough, treatments for the other likely causes were added serially to that of the first. In patients in whom no clear diagnosis could be made at the initial assessment empirical sequential therapeutic trials (STT) were carried out for the three commonest causes of cough, each over a period of 8 weeks. Treatment recommendations were along the lines of the British Thoracic Society guidelines [1]. When therapeutic trials failed, investigations were carried out as per the investigational protocol described below. Investigations were also carried out when initial screening had suggested specific lung pathology.

2. ***Investigational protocol***

Patients who failed to respond to treatment in the clinical protocol were managed by an investigational protocol similar to that used by most secondary referral centres for investigation and management of cough [10,11]. Investigations requested were based on clinical suspicion. These include sputum cytology studies, bronchial provocation testing, sinus x-rays and computerised tomography (CT), chest CT, upper gastrointestinal studies (gastroesophagoscopy, oesophageal manometry and 24 hr pH monitoring) and fibreoptic bronchoscopy.

3. ***Study endpoint***

Successful response to treatment was taken as confirmation of diagnosis and the study endpoint. It was defined as treatment for the given diagnosis resulting in either cessation of cough or significant reduction in cough to a point that it no longer disturbed the patient. This was based on patients self reported subjective response.

### Analysis of data

Data analysis was performed using SPSS version 10.0 (SPSS Inc; Chicago, IL, USA). Data for patient characteristics were expressed as mean (±SD). Relationship between symptoms and diagnosis was analysed using Fisher’s Exact t-test with level of significance taken as p = 0.05. The sensitivity and specificity of symptoms in directing diagnosis was compared in patients in whom treatment was successful based on initial assessment to those who either needed STT or investigations for diagnosis. Data was analysed on an intention to treat basis.

### Ethical approval

Ethical approval for this study was granted by the Hull and East Riding Local Research Ethics Committee.

## Results

One hundred and twelve (73 female, mean age 56.2 years) patients were recruited into this study. Table 1 summarises the main characteristics of patients at the time of enrolment in the study. Eighty one patients were managed in the clinical arm. Of these, 74 (66%) were discharged following a successful response to treatment and 7 were lost to follow up. Thirty one (27.7%) patients were converted to the investigational protocol after failure of response to treatment in the clinical protocol. In 18 of these a diagnosis was made, 8 remain undiagnosed and 5 were lost to follow-up.

**Table 1:** Patient characteristics at the time of referral

**Total number of patients (number female)**	**112 (73)**
Mean age	56.2 years
Median duration of cough (range)	3 years (0.25-64 years)
Smoking history:	
Current smokers	6 (5%)
Ex-smokers	46 (41%)
Never smoked	60 (54%)
Sputum production:	
No sputum	45 (40%)
Clear sputum	43 (38%)
Purulent sputum	23 (21%)
Cough preceded by URTI	35 (31%)
Symptoms associated with cough:	
Wheeze	46 (41%)
Breathlessness	60 (54%)
Acid taste in mouth	26 (23%)
Heartburn	48 (43%)
PND	40 (36%)
Throat clearing	73 (65%)

URTI = upper respiratory tract infection, PND = post-nasal drip. Cough duration did not have a normal distribution.

### Clinical protocol results

In 51 (45.5%) patients, therapy based on the differential diagnosis at initial assessment led to resolution or significant reduction of symptoms, while 23 (20.5%) patients responded to STT. Reflux related cough and asthma comprised more than two thirds of the initial diagnoses considered. Therapy prescribed is as per Figure 1. On STT a majority of patients responded to treatment for the second diagnosis instituted as part of the STT. The commonest single cause of cough was reflux related cough. The triad of reflux related cough, asthma and chronic rhinitis comprised the vast majority (Table 2). Mean duration of follow-up for those in the clinical protocol was 16.9 (±10.8) weeks. Mean subjective cough severity as recorded on the numeric response score (Additional file 1) improved from 6.3 (±2.5) to 2.7 (±2.8) (p < 0.0001).

**Table 2:** Diagnoses of patients at discharge

**Diagnosis**	**Clinical Assessment**	**Sequential Therapeutic Trials**	**Conventional Protocol**	**Total**
	**n** = **51**	**n** = **23**	**n** = **18**	**n** = **92**
	**Frequency(%)**	**Frequency(%)**	**Frequency(%)**	**Frequency(%)**
Reflux related cough	19 (37.3)	2 (8.7)	6 (33.3)	27 (29.3)
Asthma	17 (33.3)	2 (8.7)	1 (5.6)	20 (21.7)
Rhinitis	5 (9.8)	3 (13)	3 (11.7)	11 (12)
Post-infective	4 (7.8)	0	0	4 (4.3)
Multiple Causes	3 (5.8)	8 (34.7)	0	11 (12)
Idiopathic	0	0	7 (38.9)	7 (7.6)
Other *	3 (5.8)	8 (34.7)	1 (5.6)	12 (13)

The clinical assessment arm includes those who responded to treatment for the initial diagnosis made at the first clinic review. The sequential therapeutic trials arm comprised those responded to treatment given for an alternate diagnosis, having failed therapy for the initial diagnosis considered. The investigational protocol arm comprised those failed to respond to sequential therapeutic trials and needed specific investigations to establish a diagnosis. A diagnosis of post infective cough was made on the basis of a history to suggest a preceding respiratory tract infection and the cough having improved at the clinic review. Other* diagnoses included bronchiectasis, cancer, interstitial lung disease and self-limiting cough.

Two patients had chest x-rays suggestive of lung cancer and appropriate investigations confirmed this. Chest CT scans were also performed in the patients with bronchiectasis and interstitial lung disease as part of the management process.

### History and basic investigations

In the 51 patients in whom successful treatment was based on the initial assessment, symptoms associated with diagnosis of reflux associated cough were heartburn and sour taste in the mouth (p ≤ 0.01). A diagnosis of asthma was associated with a history of wheeze and dyspnoea (p ≤ 0.01). Rhinitis was significantly associated with postnasal drip (p = 0.002). The amount and type of phlegm did not contribute to the diagnosis. Examination findings were helpful in patients with bronchiectasis and interstitial lung disease. Reversibility was highly specific but poorly sensitive for the diagnosis of asthma. Chest radiograph was contributory in 7 (6.3%) patients. The sensitivity and specificity of various symptoms related to final outcomes are shown in Table 3.

**Table 3:** Sensitivity and specificity of symptoms

**Diagnosis**	**Symptom**	**Sensitivity %**	**Specificity %**	**p value**
**Asthma**	Wheeze	94	66	<0.001
	Breathlessness	82	51	0.009
	Airways obstruction (FEV_1_/FVC < 70%)	35	80	0.07
	Reversibility	11	95	0.2
**Reflux**	Acid taste	50	80	0.01
	Heartburn	72	68	0.001
**Rhinitis**	Post nasal mucus drip	100	67	0.002
	Throat clearing	100	37	0.07

## Discussion

Our results indicate that it is possible to successfully manage a majority of patients with chronic cough based on history, examination and simple tests. Patients in this study had all been seen previously by at least one physician and included tertiary referrals. A combination of treatment of the likely diagnoses and sequential therapeutic trials for the commonest causes of cough led to resolution of symptoms in two-thirds of our patients. This in fact compares favourably with other studies that have used an investigational approach in management of cough patients [4,12,13]. Our results suggest that it would be possible to successfully manage a significant proportion of patients with chronic cough without the need for specialist referral or investigations. This is a management approach which is both effective and cost saving.

A previous study looking into managing patients using an approach of a sequence of trial-and-error treatments based on a presumptive hierarchy of possible diagnoses has shown that this is a feasible option [14]. In this study chronic cough was defined as that lasting more than 4 weeks and a trial of treatment of a week was much shorter than in our study. The commonest cause for chronic cough, based on response to treatment, was found to be post nasal drip syndrome. Previous epidemiological studies have shown wide variation in specific aetiologies of chronic cough [7,8]. In this study a response rate of over 90% was demonstrated although arguably in view of the definition of chronic cough used, in a proportion of patients their cough may have been self limited. The results of both our study and this report support this approach to managing patients with chronic cough.

Since this study was performed in a specialist cough clinic, one might question whether the sample is representative of the general practice population. Although we know that cough is a common problem in primary care, there are few studies in the literature looking at the causes of chronic cough in this setting. Patient selection may be a problem, but in practice, the fundamental difficulty is how to confirm a diagnosis. This study’s principal aim was to test the efficacy of a diagnostic protocol, which could be used in general practice, rather than to describe the frequency of causes of chronic cough. Since approximately 75% of the patients in our sample were referred from primary care, this group should represent patients which General Practitioners find difficult to diagnose and for whom use of a clinical diagnostic protocol would be beneficial.

Confirmation of diagnosis was based on successful therapeutic trial. The most common diagnosis was GOR related chronic cough. It is well known that there is a large placebo response seen in the treatment of chronic cough. It has been shown the response in cough to acid suppression with proton pump inhibitors is no better than placebo [15,16]. Presence of dyspeptic symptoms was a marker of positive response seen in this study, which is what we have observed in our randomised trial as well [15]. Unlike the classical symptoms of GOR disease where the result of gastric acid exposure lead to mucosal damage and symptoms, non-acid or even gaseous reflux is thought to play a major role in reflux associated cough. Acid suppressive therapy leads to a decrease in gastric acid secretion but does not lead to improvement in reflux events themselves as these are mainly related to transient lower oesophageal sphincter relaxation (TLOSR) [17,18]. Further escalation for treatment for reflux used in our clinic includes a trial of pro-kinetics (metoclopramide and domperidone) and drugs which reduce TLOSR (baclofen). Baclofen is known to attenuate pharmacologically induced cough as well [19,20]. Although we do not have data from well organised clinical trials to support the above, an empirical trial is justified from our understanding of reflux related cough. Although commonly used for other indications, all the above medications do have their associated adverse effects. Metoclopramide, Domperidone and Baclofen are all associated with CNS side effects. The very commonly used proton pump inhibitors also have associated side effects which include an increased risk of developing pneumonia [15]. The benefits of a trial of therapy have to be balanced against the possible adverse effects. All our patients had chronic debilitating cough and empirical trial of treatment was discussed with them prior to initiation of therapy.

Antihistamines may have activity on a number of tussive mechanisms [21]. Central reduction of cough threshold may be associated with sedative activity, whereas blockade of mast cell derived histamine may be an effective treatment of rhinitis or asthma [22,23]. Antihistamines may also work on attenuating the cough reflex itself by an effect on the transient receptor potential ion channels [24]. Because of this lack of specificity a precise diagnosis cannot necessarily be relied on in this study. We suggest that the response to therapy experienced by patients was unlikely to be simply a placebo effect in view of the failure of previous treatment and the long duration of cough.

We assessed response to therapy based on subjective report of the patient and a graduated numeric response scale. Cough-specific quality-of-life scales have been validated in the assessment of chronic cough. Other descriptive scores or visual analogue scales have not been validated. We have shown that various forms of subjective assessment of cough are reproducible at 8 weeks and correlate well with both objective cough counting as well as cough-specific quality-of-life questionnaire scores [25]. Hence although the scale used by us in this study cannot reflect scores on other subjective means of assessment, such as the visual analogue scale, it would be appropriate to subjectively quantify the severity of cough.

As in other studies two thirds of our patients were female. GOR was more common amongst our patients while fewer had rhinitis as compared to previous studies [6,7]. All patients had previously been tried on some form of therapy and it was only when these did not succeed that they were referred to the cough clinic. This may well have altered the mix of patients we saw compared with the general population. Studies have shown that chronic cough is slow to resolve on therapy and this may explain why therapeutic trials of less than eight weeks have limited success [4,26]. In a significant proportion of patients no underlying diagnosis was established despite a sequential trials of treatment and extensive investigations. This is the experience of other centres as well. Recently the “cough hypersensitivity syndrome” has been proposed as a unifying diagnosis for patients with chronic cough which may explain the pathogenesis of cough in this group of patients [27]. Our group of patients did not have another clinic review once they had reported improvement in their cough. Hence long term follow up data is not available and is a limitation of this study.

It is of interest to note that although 31% of patients gave a history of an upper respiratory tract infection preceding the onset of the cough only 4% had a diagnosis of post-infective cough at discharge. In general, respiratory tract infections cause acute cough more commonly than chronic cough, but prolonged post viral cough does occur. This often resolves spontaneously and therefore may have been selected out of our study population in the time between referral and attendance at clinic. However, since viruses seem to cause cough by lowering the threshold of the cough reflex, [28,29] it is not uncommon in our experience to see patients in whom an upper respiratory tract infection seems to unmask a cough which is due to a different cause but was previously sub-clinical.

In contrast to previous studies which did not find information on the character, timing and complications of cough useful in identifying the underlying cause, [30] we have shown that important diagnostic information is contained in the presenting complex of symptoms which are usually associated with the common causes of cough. For instance dyspeptic symptoms, if present, would suggest a diagnosis of reflux related cough. However quite often dyspeptic symptoms are absent in patients with reflux related cough. Other symptoms which would point to this diagnosis include: cough on phonation, on rising from bed, associated with certain foods or with eating in general [31]. There is considerable overlap of associated symptoms which patients with chronic cough have, however based on the presentation a correct diagnosis can be established in a large proportion. We think that airway reflux leading to an enhanced cough reflex is the most common cause of chronic cough and we have termed this the cough hypersensitivity syndrome. The validated Hull Airway Reflux Questionnaire is a useful tool to diagnose this [27].

## Conclusion

In this study we have demonstrated that it is possible to manage a majority of chronic cough patients successfully using a clinically based protocol without the need of intensive investigations. Therapeutic trials must be of adequate duration to be successful. The presenting complex of symptoms was indicative of the underlying cause of cough highlighting the importance of history taking in the assessment of patients. Chest x-rays, despite their low sensitivity, are a useful tool in screening for parenchymal lung pathology. Spirometric results are specific but do not significantly contribute to the diagnosis of cough predominant asthma.

## Competing interests

None of the authors have any competing interests relevant to this study.

## Authors’ contributions

All the authors were involved in the management of patients seen in our cough clinic. CFE drafted the initial manuscript. All authors have read and approved the final manuscript.

## Supplementary Material

Additional file 1 Cough Clinic Proforma. Click here for file
