# Supplementary material for: Management of patients with chronic cough using a clinical protocol: a prospective observational study
Source: Cough. 2013 Jan 24;9:2. doi: 10.1186/1745-9974-9-2 (PMC3565860; doi:10.1186/1745-9974-9-2)
Supplement: Additional file 1 — Cough Clinic Proforma. [file 1745-9974-9-2-S1.doc]

**Appendix 1**

**Cough Clinic Proforma:**

(This form is to be filled by the physician. Ring answers where appropriate)

***Demographic Data:***

Date____/____/____ Patient Number: _________________________

DoB____/____/____Age ____________Sex Male/Female

***History of cough:***

Unbearable symptoms

1. Duration of cough_____________

10 -

9 -

8 -

7 -

6 -

5 -

4 -

3 -

2 -

1 -

0 -

2. Description of cough

2.1 Severity of cough :

Indicate on the adjacent scale how severe you feel your cough is. 0 is no cough at all and 10 is a continuous most disturbing cough

- 1. Characteristics of cough (intermittent/ continuous etc):

3. Was cough preceded by chest/ upper airway infection

4. Is the cough productive?

4.1. If yes, of what

Clear Phlegm Purulent Phlegm Haemoptysis

4.2. Amount of expectorate

No cough

Small (< ¼ cupful) Moderate (¼ - ½ cupful) Large (>½ cupful)

5. Does the patient have associated symptoms?

Wheeze Breathlessness Sour taste in mouth Heartburn Choking sensation Throat clearing

Nasal discharge Nasal congestion Postnasal drip

6. Are there triggering factors?

Change in temperature Exercise Aeorsols /spray

7. Is their any periodicity to the cough?

Related to eating Supine or on stooping Night > day

Day more than night Both day and night Related to work

#### Past medical history

1. Was there significant history of childhood illness?

2. Hay fever Eczema Nasal polyps

3. Any other medical condition for which the patient has received or is receiving treatment? If yes please list them down:

#### Medication

#### Family and social history

1. Smoking History:

Current smoker Ex-smoker Non-smoker Pack years

2. Alcohol intake (units/week)

3. Pets yes no

3.1 If yes what pets

4. Nuclear family history of atopy yes no

4.1 If yes specify

5. Occupational History

#### Examination

1. General examination:

1.1. HR______________ Bp_____________ Weight_________ Height_______

1.2.Clubbing 1.3 Any other significant findings:

2. Nose and throat

2.1 Pharyngeal mucus 2.2 Cobblestone appearance 2.3 Nasal congestion 2.4 Facial tenderness

2.5 Any other significant findings:

1. Respiratory system
   1. Tachypnoea
   2. Normal auscultation
   3. Impaired air entry
   4. Wheeze
   5. Crepitations
   6. Other significant findings:

4. Cardiovasular system

Normal abnormal

5. Abdominal Examination

Normal abnormal

***Pulmonary Function Tests:*** (Actual/ Predicted)

FEV1***_______*** FVC_______ FEV_1_/FVC__________

PEFR_______ Reversibility_______

***Chest x-ray***

***Diagnoses:***

Primary cause(s) of cough

Other diagnoses

***Treatment:***

#### Tests Requested

***Comments***
